# Effects of moldy corn on the performance, antioxidant capacity, immune function, metabolism and residues of mycotoxins in eggs, muscle, and edible viscera of laying hens

**DOI:** 10.1016/j.psj.2023.102502

**Published:** 2023-01-14

**Authors:** Fenghua Zhu, Lianqin Zhu, Jindong Xu, Yuchang Wang, Yang Wang

**Affiliations:** ⁎ College of Animal Science and Technology, Qingdao Agricultural University, Qingdao 266109, P.R. China; † College of Veterinary Medicine, Qingdao Agricultural University, Qingdao 266109, P.R. China; ‡ College of Science and Information, Qingdao Agricultural University, Qingdao 266109, P.R. China

**Keywords:** moldy corn, aflatoxin B1, zearalenone, deoxynivalenol, laying hen

## Abstract

Mycotoxins, including aflatoxin B1 (**AFB_1_**), zearalenone (**ZEN**) and deoxynivalenol (**DON**), are common contaminants of moldy feeds. Mycotoxins can cause deleterious effects on the health of chickens and can be carried over in poultry food products. This study was conducted to investigate the effects of moldy corn (containing AFB_1_, ZEN, and DON) on the performance, health, and mycotoxin residues of laying hens. One hundred and eighty 400-day-old laying hens were divided into 4 treatments: basal diet (Control), basal diet containing 20% moldy corn (MC20), 40% moldy corn (MC40) and 60% moldy corn (MC60). At d 20, 40, and 60, the performance, oxidative stress, immune function, metabolism, and mycotoxin residues in eggs were determined. At d 60, mycotoxin residues in muscle and edible viscera were measured. Results showed the average daily feed intake (**ADFI**) and laying performance of laying hens were decreased with moldy corn treatments. All the moldy corn treatments also induced significant oxidative stress and immunosuppression, reflected by decreased antioxidase activities, contents of cytokines, immunoglobulins, and increased malonaldehyde level. Moreover, the activities of aspartate aminotransferase and alanine transaminase were increased by moldy corn treatments. The lipid metabolism was influenced in laying hens receiving moldy corn, reflected by lowered levels of total protein, high density lipoprotein cholesterol, low density lipoprotein cholesterol, total cholesterol, and increased total triglyceride as well as uric acid. The above impairments were aggravated with the increase of mycotoxin levels. Furthermore, AFB_1_ and ZEN residues were found in eggs, muscle, and edible viscera with moldy corn treatments, but the residues were below the maximum residue limits. In conclusion, moldy corn impaired the performance, antioxidant capacity, immune function, liver function, and metabolism of laying hens at d 20, 40, and 60. Moldy corn also led to AFB_1_ residue in eggs at d 20, 40, and 60, and led to both AFB_1_ and ZEN residues in eggs at days 40 and 60, and in muscle and edible viscera at d 60. The toxic effects and mycotoxin residues were elevated with the increase of moldy corn levels in feed.

## INTRODUCTION

Mycotoxins are natural small-molecule contaminants produced as secondary metabolites by fungi (Steyn, 1995). Contamination by mycotoxins occurs frequently in chicken feed, including maize and other cereals (Iqbal et al., 2014). Economic losses due to mycotoxin contamination occur at all levels of food and feed production including crop and animal production, processing and distribution (Galvano et al., 2001). According to a 10-year survey, in 41.1, 38.5, and 20.9% of feed and feed raw materials (e.g., maize, wheat, soybean) samples from South Asia, Sub-Saharan Africa, and Southeast Asia, respectively, the AFB_1_ contents exceeded the maximum level (20 μg/kg). DON and ZEN concentrations were also correlated in maize (Gruber-Dorninger et al., 2019). In addition, from yr 2016 to 2017, the positive detection rates for AFB_1_, ZEN, and DON in maize samples collected from 21 provinces in China were 96.1, 92.05, and 98.15%, respectively, and these 4 toxins were detected at the same time in 92.25% of the samples (Ma et al., 2018). Aflatoxins (**AF**), produced by *Aspergillus* spp., are known to have strong hepatotoxic and carcinogenic effects and are regulated by feed/food law in at least 100 countries (van Egmond and Jonker, 2004). Among the various types of AF, aflatoxin B1 (**AFB_1_**) is most commonly encountered and it is also considered to have higher toxicity than other aflatoxins (Yunus et al., 2011). To date, the most frequently found mycotoxins are deoxynivalenol (**DON**) and its acetylated derivatives, which can affect animal and human health causing acute temporary nausea, vomiting, diarrhea, abdominal pain, headache, dizziness, and fever (Sobrova et al., 2010). Moreover, zearalenone (**ZEN**) resembles the structure of naturally occurring estrogens. This property of ZEN determines its ability to bind to estrogen receptors, exerting estrogenic and anabolic effects on animals (Rogowska et al., 2019). Besides, reports have indicated that AFB_1_, DON, and ZEN can also impair the immune function, antioxidant ability and biochemical metabolism. For example, Rajput et al. (2017) demonstrated that AFB_1_ significantly influenced the serum biochemical parameters, decreased immunoglobulin (**Ig**) levels, and reduced antioxidase activities of chicken. Azizi et al. (2021) revealed that dietary DON feeding to chickens caused an enhancement in blood aspartate transaminases (**AST**) content and a decrease in blood total protein (**TP**). The antioxidase activities and antioxidant gene expressions of chickens were also reduced by DON treatment (Yang et al., 2017). Additionally, both of the humoral and cellular immunity can be impaired by DON (Li et al., 2014). As for the ZEN, Xu et al. (2021) found that ZEN treatment increased activities of AST and alanine transaminase (**ALT**), decreased antioxidase capacity, and elevated inflammatory cytokine gene expressions of chickens.

Human can also easily be exposed to mycotoxins through foods of plant origin (cereal grains) or foods of animal origin (muscle meat, milk, egg) (Wang et al., 2018). Mycotoxin residue has been found in eggs, muscle, and organs and its concentration is high in liver, egg, kidney, and thigh region (Herzallah, 2013). Fernández et al. (1994) found 0.05 μg/kg residue of AFB_1_ in muscle of laying hen exposed to 5 mg/kg AF-contaminated feed. Feeding birds diet contaminated with AF (123.0 μg/kg), or AF and ZEN (123.0 and 260.2 μg/kg, respectively) resulted in the AF residues in eggs between 0.01 and 0.21 μg/kg (Jia et al., 2016). Although in some cases low-levels of mycotoxin contamination may not affect animal growth or performance, mycotoxins may be carried over into animal liquid and tissue products (Fowler et al., 2015). Aly and Anwer (2009) showed that the egg production and weight were not affected in laying birds fed 25 to 100 μg AF/kg feed, yet there was residue in eggs. Mycotoxin residues poses a risk to food security and safety because increased residues in animal products cause economic losses through border rejection in the global and local market ultimately aggravating the macro- and micronutrient insecurity especially in developing countries (Adegbeye et al., 2020).

In order to provide more in vivo evidence for the toxicity and residue of mycotoxins in laying hens, different percentage of moldy corn were fed to chickens for 20, 40, and 60 d. At the endpoints, the performance, antioxidant ability, immune function, metabolism, and mycotoxin residues were investigated. Overall, the results of our study are helpful to raise awareness of the health risks associated with these toxins.

## MATERIALS AND METHODS

### Diets

For the preparation of moldy corn, plastic film and insulation pad were used to cover the corn. Water was sprayed once a week and the corn was stirred evenly. The corn was mildewed under natural conditions at 14 to 20°C and 65 to 70% humidity for 30 d. After 30 days mildew, the contents of AFB_1_, DON, ZEN, fumonisin (**FB**) and T-2 were measured to be 125 μg/kg, 1,220 μg/kg, 4.9 mg/kg, 2.37 mg/kg, and 45 μg/kg, respectively, by using UPLC-MS/MS. Then, 0.3% calcium propionate and sweetener were added to inhibit mildew and mask odor. The prepared moldy corn was used to replace normal corn in the basal diet for *Fusarium* mycotoxin-contaminated diet preparation. The composition and nutrient levels of the basal diet was listed in Table 1.

**Table 1:** Composition and calculated nutrient content of basal diets (air dry basis).

Ingredients	Content	Nutrients^2^	Content
Corn, %	62.50	ME, MJ/kg	10.86
Soybean meal, %	24.00	CP, %	15.70
Limestone, %	8.50	AP, %	0.48
Premix^1^, %	5.00	Ca, %	3.70
Total, %	100.00	Cl, %	0.19
		Na, %	0.30
		Lys, %	0.80
		Met, %	0.32
		Cys, %	0.33

1 The premix provided the following per kg of diets: VA 11,000 IU, VD_3_ 600 IU, VE 20 IU, VK_3_ 2 mg, VB_1_ 1.6 mg, VB_2_ 4.5 mg, VB_6_ 5 mg, VB_12_ 0.03 mg, folic acid 2 mg, pantothenic acid 10 mg, biotin 0.1 mg, choline 50 mg, Cu 10 mg, Fe 35 mg, Zn 50 mg, Mn 60 mg, Se 0.30 mg, I 0.5 mg, limestone 0.175 g, calcium dihydrogen phosphate 0.6 g, Lys 25 mg, Met 40 mg, sodium bicarbonate 0.15 g.

2 All nutrient contents were calculated.

### Experimental Design

**Figure 1:**
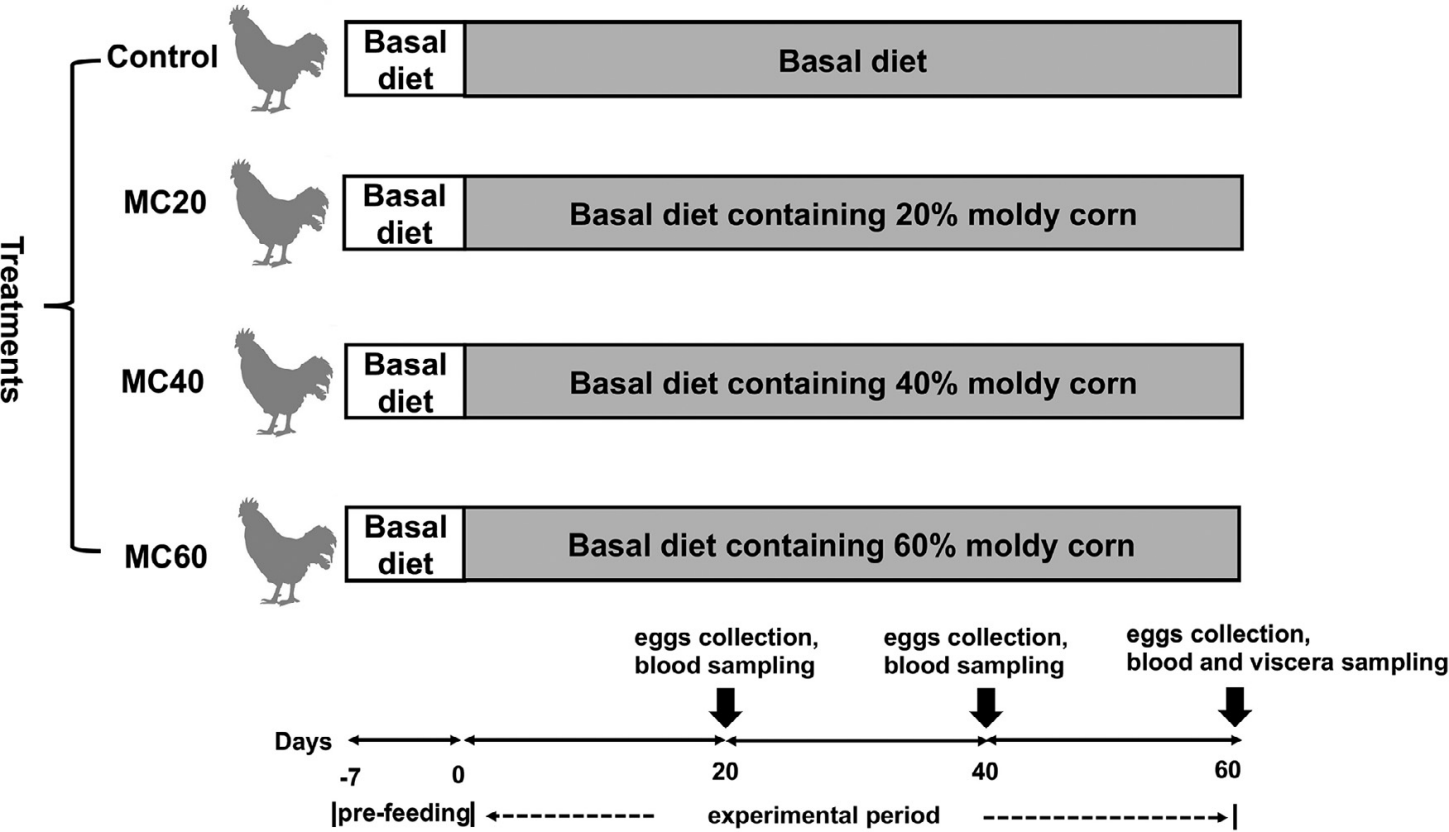
Experimental design.

One hundred and eighty 400-day-old Hy-Line Brown laying hens were reared in three-layer stepped cage and equally divided into 4 groups (Control, MC20, MC40, and MC60) with 5 replications of 9 birds each for a 7-d pre-feeding period and 60-d formal period. Three birds were reared separately in a single cage (0.47 m × 0.36 m × 0.38 m). Laying hens in the Control, MC20, MC40, and MC60 groups were fed with the basal diet, basal diet containing 20% moldy corn, 40% moldy corn, and 60% moldy corn, respectively (Figure 1). Fresh water and feed were provided ad libitum. The temperature of the room was set at 21°C. The animal experiment was approved and performed in accordance with the guidelines of Ethics and Animal Welfare Committee of Qingdao Agricultural University.

### Measurement of Performance

On a replicate basis, the feed intake, number of eggs, egg weight, number of broken eggs, and number of dead hens were recorded daily. And the average daily feed intake (**ADFI**), average egg weight, laying rate, feed/egg ratio, broken egg rate, and death/culling rate were calculated. ADFI = total daily feed consumption/number of hens; average egg weight = average daily total egg weight/total number of eggs; laying rate = (total number of eggs/laying hens number/days) × 100%; broken egg rate = (number of broken eggs//total number of eggs) × 100%; feed/egg = ADFI/(average daily total egg weight/number of hens); death/culling rate = number of dead hens/total number of hens.

### Sample Collection

At d 20, 40, and 60, one hen of each cage (3 hens of each replicate) were randomly selected and euthanized and blood samples were collected from the wing vein into vacuum tubes containing an anticoagulant and centrifuged for 10 min (3,000 *g*) at 4°C. Pure plasma samples were collected and stored in 1.5 mL Eppendorf tubes at −20°C. At d 20, 40, and 60, three eggs from each replicate were collected, mixed into one sample and dried in the oven at 60°C. Thereafter, dried eggs were smashed and passed through 20 mesh sieves. Egg samples were then collected and stored at −20°C for further detection of mycotoxin residues. At d 60, the liver, breast muscle, leg muscle, and heart from 1 hen of each cage were collected for the detection of mycotoxin residues (Figure 1).

### Analysis of Biochemical Indices in Plasma

Commercial kits (Nanjing Jiancheng Bioengineering Institute, Nanjing, China) were used to measure the activities of superoxidase (**SOD**; cat. no. A001-3-2), glutathione peroxidase (**GSH-Px**; cat. no. A005-1-2), AST (cat. no. C010-2-1), and ALT (cat. no. C009-2), and the levels of total antioxidant capacity (T-AOC; cat. no. A015-1-2), malonaldehyde (**MDA**; cat. no. A003-1), IgA (cat. no. H108-1-1), IgG (cat. no. H106-1-1), IgM (cat. no. H109-1-1), interleukin 1β (**IL-1β**; cat. no. H002), IL-6 (cat. no. H007-1-1), tumor necrosis factor α (**TNF-α**; cat. no. H052-1), TP (cat. no. A045-1-1), total cholesterol (**T-CHO**; cat. no. A111-1-1), total triglycerides (**TG**; cat. no. A110-1-1), high density lipoprotein cholesterol (**HDL-C**; cat. no. A112-1-1), low density lipoprotein cholesterol (**LDL-C**; cat. no. A113-1-1), and uric acid (**UA**; cat. no. C012-2-1) were detected spectrophotometrically according to manufacturer's protocol.

### Measurement of Mycotoxins

The contents of mycotoxins in the feed, muscle, edible viscera, and eggs were measured using UPLC-MS/MS (Agilent Technologies). Briefly, 10 mL of 0.05 mol/L sodium acetate (pH = 4.8) and 0.025 μL glucuronidase/sulfate complex enzyme were added in 3 g samples, vortex-mixed and vibrated in gas bath at 37°C for 12 h. Then, 10 mL acetonitrile (containing 2% formic acid), 4 g MgSO_4_ and 1.5 g NaCl were added, and the sample were extracted by vibration for 2 h. After centrifuge at 5,000 r/min for 5 min, the upper acetonitrile phase (8 mL) was pipetted into a 10 mL tube and diluted with 2 mL water and vortex-mixed. The extract (2.5 mL) was loaded into a 3 mL Captiva EMR-Lipid purification tube (Agilent Technologies, Santa Clara, California) and allowed to flow through the purification tube by gravity. After the extract was completely eluted, vacuum was applied to evacuate the purification tube. Transfer 0.5 mL eluate to an autosampler tube and add 0.3 mL of 0.1% formic acid in 5 mmol/L ammonium formate. The mixed solution was filtered through a 0.22-μm membrane filter and injected into the UPLC-MS/MS system for analysis.

Chromatographic separation was carried out using a 50 mm × 2.1 mm i.d., 2.7 μm, C18 column (Waters, Milford, MA). A 5 mmol/L ammonium formate containing 0.1% formic acid was used as the mobile phase at a flow rate of 0.3 mL min^−1^. The column temperature was maintained at 40°C and the injection volume was 5 μL. The UPLC system was coupled to a triple quadrupole tandem mass spectrometer (TQ Detector; Waters) with an electrospray ion source (**ESI**). The MS detector was operated in positive-ion mode with the following settings: capillary voltage, 3.5 kV; source temperature, 120°C; desolvation temperature, 350°C; desolvation gas flow, 650 l h^−1^; and cone gas flow, 50 l h^−1^. High purity nitrogen was used as the collision gas, and the collision gas flow was 0.20 mL min^−1^. Cone voltage and collision energy were optimized by infusion of each individual analyte. Detection was carried out in multiple reaction monitoring (**MRM**) mode. MRM parameters for MS detection of the 3 mycotoxins are summarized in Table S1.

### Method Validation

The validation parameters considered were linearity (**R^2^**), recovery rate, precision (**RSD**), and limit of detection (**LOD**) and quantification (**LOQ**). Recovery rate and RSD were evaluated via blank samples spiked at different concentrations. Calculations were based on relative peak areas, that is, the peak area of the compound of interest divided by the peak area of the corresponding IS added to the same sample (Emmanuel et al., 2020).

As for the validation results, the upper limit of recovery rate varied between 92.2% and 112.7% for egg, liver, heart, leg muscle, and breast muscle. The lower limit of recovery rate varied between 80.5% and 99% for egg, heart, leg muscle, and breast muscle. RSD were found to be adequate for the different mycotoxins (Table S2). The LOD values for AFB_1_, ZEN and DON were 0.0015 μg/kg, 0.06 μg/kg, and 2.7 μg/kg, respectively. The LOQ values for AFB_1_, ZEN, and DON were 0.0045 μg/kg, 0.20 μg/kg, and 9.13 μg/kg, respectively (Table S3). The criteria of linearity (R^2^ ≥ 0.997) were fulfilled for all the mycotoxins studies.

### Statistical Data Analysis

All data were subjected to analysis of variance (moldy corn percentage) as an entirelyrandomized design using the general linear model (**GLM**) of SAS (SAS Institute Inc, version 9.3, Cary, North Carolina), followed by Duncan's multiple comparisons test. Results were presented as the means ± standard deviation (**SD**). The differences were considered significant at *P* < 0.05.

## RESULTS

### Contents of AFB_1_, ZEN, and DON in the Experimental Diets

The AFB_1_, ZEN, DON and FB levels in the diets of Control group were 0.10 μg/kg, 51.7 μg/kg, 0.20 mg/kg, and 1.70 mg/kg, respectively, which were all below the mycotoxin limit standard of China. The AFB_1_, ZEN, DON, FB, and T-2 levels in the diets of MC20 group were 25.36 μg/kg, 245.05 μg/kg, 0.99 mg/kg, 0.49 mg/kg, and 9 μg/kg, respectively, and the AFB_1_ level was higher than the mycotoxin limit standard of China. The AFB_1_, ZEN, DON, FB, and T-2 levels in the diets of MC40 group were 51.62 μg/kg, 505.73 μg/kg, 2.12 mg/kg, 0.96 mg/kg, and 18 μg/kg, respectively. The AFB_1_ and ZEN levels were higher than the mycotoxin limit standard of China. Moreover, the AFB_1_, ZEN, DON, FB and T-2 levels in the diets of MC60 group were 76.50 μg/kg, 750.30 μg/kg, 3.34 mg/kg, 1.43 mg/kg, and 27 μg/kg, and levels of AFB_1_, ZEN, and DON were all higher than the mycotoxin limit standard of China (Table 2). Thus, by using these experimental diets, the toxic effects of moldy corn can be investigated.

**Table 2:** Mycotoxins contents in moldy feed.

	Control	MC20	MC40	MC60	Mycotoxin limit standard (GB 13078-2017, China)
AFB_1_ (μg/kg)	0.10	25.36	51.62	76.5	20
ZEN (μg/kg)	51.70	245.05	505.73	750.3	500
DON (mg/kg)	0.20	0.99	2.12	3.34	3
FB (mg/kg)	1.70	0.49	0.96	1.43	20
T-2 (μg/kg)	Not detected	9.00	18.00	27.00	500

Abbreviations: AFB_1_, aflatoxin B1; ZEN, zearalenone; DON, deoxynivalenol; FB, fumonisin; MC20, basal diet containing 20% moldy corn; MC40, basal diet containing 40% moldy corn; MC60, basal diet containing 60% moldy corn.

### Effects of Moldy Corn on the Performance of Laying Hens

From d 1 to d 20, the ADFI was significantly decreased in all the moldy corn treatments (*P* < 0.05) and the value in MC60 group was lower than that of the MC20 and MC40 groups (*P* < 0.05). The laying rate was significantly decreased by all the moldy corn treatment groups (*P* < 0.01) in a dose-dependent manner. The average egg weight was also lowered in the MC60 group compared to other groups (*P* < 0.05). The broken egg rate was significantly elevated in the MC60 group compared to the Control and MC20 groups (*P* < 0.01).

From d 1 to d 40, the ADFI was significantly decreased all the moldy corn treatments (*P* < 0.05), and the value in MC60 group was lower than that of the MC20 and MC40 groups (*P* < 0.01). The laying rate was also significantly decreased in all the moldy corn treatments (*P* < 0.05) in a dose-dependent manner. The average egg weight and feed/egg ratio were significantly reduced in the MC40 and MC60 groups (*P* < 0.05). The MC60 treatment led to elevated broken egg rate (*P* < 0.01), which was higher than that of the MC20 (*P* < 0.01) and MC40 (*P* < 0.05) groups.

Moreover, from d 1 to d 60, the ADFI, laying rate, and average egg weight were significantly decreased in all the moldy corn treatments (*P* < 0.05) in a dose-dependent manner. The feed/egg ratio was significantly increased in the MC40 and MC60 groups (*P* < 0.01), and the MC60 treatment led to a higher feed/egg ratio than that of the MC20 treatment (*P* < 0.05). The broken egg rate was significantly elevated in all the moldy corn treatments (*P* < 0.05), and was higher in MC60 treatment than in that of the MC20 and MC40 groups (*P* < 0.01). The death/culling rate was not significantly altered among groups (*P* > 0.05; Table 3).

**Table 3:** Effects of moldy corn on the laying performance of laying hens.

days	Items	Treatments				*P* value
		Control	MC20	MC40	MC60	
20	ADFI, g	112.56 ± 4.61^A^^a^	104.27 ± 4.36^AB^^b^	97.73 ± 7.19^BC^^b^	87.39 ± 6.26^C^^c^	< 0.0001
	Laying rate, %	88.22 ± 2.68^A^	82.22 ± 2.22^B^^a^	76.44 ± 3.68^BC^^b^	71.33 ± 3.72^C^^c^	< 0.0001
	Average egg weight, g	62.68 ± 0.68^A^	61.77 ± 0.65^A^	61.29 ± 0.65^AB^^a^	58.65 ± 2.76^B^^b^	0.004
	Feed/egg	2.03 ± 0.09	2.05 ± 0.07	2.09 ± 0.19	2.09 ± 0.13	0.866
	Broken egg rate, %	0.00 ± 0.00^B^	0.28 ± 0.38^B^	0.59 ± 0.63^AB^	1.23 ± 0.64^A^	0.007
	Death/culling rate, %	0.00 ± 0.00	0.00 ± 0.00	0.00 ± 0.00	0.00 ± 0.00	-
40	ADFI, g	110.67 ± 5.47^A^^a^	103.09 ± 4.41^AB^^b^	97.79 ± 3.84^B^	87.66 ± 4.62^C^	< 0.0001
	Laying rate, %	84.33 ± 1.59^A^^a^	80.22 ± 3.46^AB^^b^	77.89 ± 1.98^BC^^b^	73.78 ± 1.27^C^^c^	< 0.0001
	Average egg weight, g	63.10 ± 0.82^A^	59.94 ± 2.74^AB^	57.89 ± 2.61^BC^^a^	53.39 ± 3.64^C^^b^	0.000
	Feed/egg	2.08 ± 0.10^b^	2.15 ± 0.04^ab^	2.17 ± 0.04^a^	2.23 ± 0.06^a^	0.015
	Broken egg rate, %	0.13 ± 0.29^B^	0.29 ± 0.39^B^	0.71 ± 0.49^AB^^b^	1.67 ± 1.00^A^^a^	0.004
	Death/culling rate, %	0.00 ± 0.00	0.00 ± 0.00	0.00 ± 0.00	0.00 ± 0.00	-
60	ADFI, g	107.80 ± 1.61^A^^a^	101.26 ± 3.53^AB^^b^	95.02 ± 2.08^BC^^c^	87.54 ± 7.44^C^^d^	0.0001
	Laying rate, %	83.22 ± 2.40^A^^a^	79.22 ± 2.88^AB^^b^	75.33 ± 2.06^BC^^c^	70.78 ± 2.50^C^^d^	0.0001
	Average egg weight, g	59.81 ± 1.86^A^	54.39 ± 2.14^B^	50.79 ± 1.97^C^	47.05 ± 1.41^D^	0.0001
	Feed/egg	2.17 ± 0.11^B^	2.36 ± 0.17^AB^^b^	2.49 ± 0.14^A^^ab^	2.63 ± 0.19^A^^a^	0.002
	Broken egg rate, %	0.13 ± 0.30^C^^b^	0.98 ± 0.39^BC^^a^	1.18 ± 0.65^B^	2.35 ± 0.53^A^	0.0001
	Death/culling rate, %	0.00 ± 0.00	0.00 ± 0.00	2.22 ± 4.97	4.44 ± 6.09	0.261

Abbreviations: ADFI, average daily feed intake; MC20, basal diet containing 20% moldy corn; MC40, basal diet containing 40% moldy corn; MC60, basal diet containing 60% moldy corn.

Results are represented as mean values ± SD (n = 5 per group).

a,b,c,d Mean value within a role with no common superscript differ significantly (*P* < 0.05).

A,B,C,D Mean value within a role with no common superscript differ very significantly (*P* < 0.01).

### Effects of Moldy Corn on the Antioxidant Capacity of Laying Hens

At d 20, the T-AOC level was significantly decreased by the MC40 and MC60 treatments (*P* < 0.05). The SOD activity was significantly decreased by all the moldy corn treatments (*P* < 0.01), and was lower in the MC60 group than that in the MC20 and MC40 groups (*P* < 0.01). The GSH-Px activity was significantly decreased in all the moldy corn treatments (*P* < 0.05), and was lower in the MC60 group than that in the MC20 group (*P* < 0.05). The MDA content was significantly increased in MC40 and MC60 groups (*P* < 0.01) in a dose-dependent manner.

At d 40, the T-AOC level and SOD activity were significantly decreased in the MC40 and MC60 groups (*P* < 0.05). The GSH-Px activity was significantly lowered (*P* < 0.01), while the MDA content was significantly increased in all the moldy corn treatment groups (*P* < 0.01) in a dose-dependent manner.

At d 60, the T-AOC level and SOD activity were significantly decreased in all the moldy corn treatments in a dose-dependent manner. The GSH-Px activity was also significantly decreased by all the moldy corn treatments (*P* < 0.01), and was lower in the MC60 group than in the MC20 (*P* < 0.01) and MC40 (*P* < 0.05) groups. Conversely, all the moldy corn treatments led to significantly increased MDA content (*P* < 0.01), which reached the highest level in MC60 group (Table 4).

**Table 4:** Effects of moldy corn on the antioxidant capacity of laying hens.

days	Items	Treatments				*P* value
		Control	MC20	MC40	MC60	
20	T-AOC (mmol/L)	6.29 ± 0.33^a^	6.25 ± 0.10^ab^	6.02 ± 0.07^bc^	5.91 ± 0.15^c^	0.017
	SOD (U/mL)	241.13 ± 6.63^A^	224.27 ± 6.60^B^	219.05 ± 5.18^B^	206.63 ± 6.49^C^	< 0.0001
	GSH-Px (U/mL)	81.77 ± 5.71^A^^a^	74.80 ± 5.06^AB^^b^	70.47 ± 4.89^B^^bc^	68.10 ± 2.47^B^^c^	0.002
	MDA (nmol/mL)	5.32 ± 0.07^C^	5.42 ± 0.05^C^	5.61 ± 0.07^B^	6.01 ± 0.09^A^	< 0.0001
40	T-AOC (mmol/L)	6.22 ± 0.08^A^	6.11 ± 0.24^AB^^a^	5.87 ± 0.10^BC^^b^	5.76 ± 0.10^C^	0.000
	SOD (U/mL)	253.80 ± 7.33^A^	245.30 ± 7.51^AB^^a^	235.90 ± 7.84^BC^^b^	225.59 ± 3.97^C^^c^	< 0.0001
	GSH-Px (U/mL)	80.11 ± 2.22^A^	75.04 ± 1.73^B^	73.50 ± 3.16^BC^^a^	69.08 ± 3.09^C^^b^	< 0.0001
	MDA (nmol/mL)	5.82 ± 0.12^C^	6.21 ± 0.11^B^^b^	6.45 ± 0.17^B^^a^	6.94 ± 0.17^A^	< 0.0001
60	T-AOC (mmol/L)	6.18 ± 0.10^A^^a^	6.02 ± 0.08^Ab^	5.72 ± 0.08^B^^c^	5.58 ± 0.11^B^^d^	< 0.0001
	SOD (U/mL)	270.28 ± 2.53^A^^a^	263.18 ± 6.74^A^^b^	252.45 ± 6.19^B^	240.34 ± 3.93^C^	< 0.0001
	GSH-Px (U/mL)	81.07 ± 1.98^A^	75.62 ± 2.01^B^	73.48 ± 1.94^BC^^a^	70.51 ± 1.24^C^^b^	< 0.0001
	MDA (nmol/mL)	6.30 ± 0.08^C^	6.87 ± 0.09^B^	7.08 ± 0.08^Ab^	7.21 ± 0.11^A^^a^	< 0.0001

Abbreviations: MDA, malondialdehyde; MC20, basal diet containing 20% moldy corn; MC40, basal diet containing 40% moldy corn; MC60, basal diet containing 60% moldy corn; GSH-Px, glutathione peroxidase; SOD, superoxide dismutase; T-AOC, total antioxidant capacity.

Results are represented as mean values ± SD (n = 15 per group).

a,b,c Mean value within a role with no common superscript differ significantly (*P* < 0.05).

A,B,C Mean value within a role with no common superscript differ very significantly (*P* < 0.01).

### Effects of Moldy Corn on the Immune Function of Laying Hens

At d 20, the IFN-α and IL-1β concentrations were significantly decreased by all the moldy corn treatments (*P* < 0.01), and were lower in the MC60 group than that in the MC20 and MC40 groups (*P* < 0.05). The IL-6 and IgA concentrations were significantly reduced in the MC40 and M60 groups (*P* < 0.01) in a dose-dependent manner. The IgG concentration was significantly reduced by all the moldy corn treatments (*P* < 0.01) in a dose-dependent manner. Moreover, the IgM concentration in the MC60 group was much lower than that of other groups (*P* < 0.05).

At d 40, the IFN-α and IL-1β concentrations were significantly decreased by all the moldy corn treatments (*P* < 0.05), and were lower in the MC40 and MC60 groups than in MC20 group (*P* < 0.05). The concentrations of IL-1β, IL-6, IgA, and IgG were also decreased by all the moldy corn treatments (*P* < 0.01). The IL-1β concentration in the MC40 and MC60 groups were lower than that of the MC20 group (*P* < 0.01). The IL-6 and IgA concentrations were decreased in a dose-dependent manner. The IgG concentration in the MC60 group was lower than that of MC20 (*P* < 0.01) and MC40 (*P* < 0.05) groups. Besides, the IgM concentration was reduced by MC40 (*P* < 0.05) and MC60 (*P* < 0.01) treatments.

At d 60, the concentrations of IFN-α, IL-1β, IL-6, IgA, and IgG were significantly reduced in all the moldy feed treatment groups (*P* < 0.01). Moreover, the IFN-α concentration of MC60 group was lower than that of the MC20 (*P* < 0.01) and MC40 (*P* < 0.05) groups. The IL-1β concentration in the MC40 and MC60 groups was lower than that of the MC20 group (*P* < 0.01). Moreover, all the moldy corn treatments decreased IL-6 (*P* < 0.01) and IgA (*P* < 0.05) concentrations in a dose-dependent manner. The IgG concentration was decreased in the MC60 group compared to that of the MC20 group (*P* < 0.01). Additionally, the IgM concentration was reduced in the MC40 and MC60 groups (*P* < 0.01) in a dose-dependent manner (*P* < 0.05; Table 5).

**Table 5:** Effects of moldy corn on the immune function of laying hens.

days	Items	Treatments				*P* value
		Control	MC20	MC40	MC60	
20	IFN-α (pg/mL)	189.36 ± 12.15^A^	163.15 ± 14.12^B^^a^	157.83 ± 15.32^B^^a^	140.12 ± 10.69^B^^b^	0.000
	IL-1β (ng/L)	22.32 ± 1.12^A^	18.56 ± 1.22^B^	17.39 ± 1.53^BC^^a^	15.22 ± 1.63^C^^b^	< 0.0001
	IL-6 (ng/L)	25.12 ± 0.50^A^	24.22 ± 1.29^A^	20.27 ± 0.32^B^	15.87 ± 0.30^C^	< 0.0001
	IgA (μg/mL)	10.73 ± 0.92^A^	9.55 ± 0.89^A^	7.63 ± 0.77^B^^a^	6.44 ± 0.93^B^^b^	< 0.0001
	IgG (μg/mL)	98.24 ± 0.98^A^	92.15 ± 1.35^B^	82.32 ± 1.67^C^^a^	79.88 ± 1.22^C^^b^	< 0.0001
	IgM (ng/mL)	5.82 ± 0.32^A^	5.67 ± 0.28^A^	5.55 ± 0.26^AB^^a^	4.96 ± 0.45^B^^b^	0.004
40	IFN-α (pg/mL)	181.42 ± 14.25^A^^a^	160.25 ± 13.23^AB^^b^	146.52 ± 12.32^Bb^^c^	139.26 ± 14.25^B^^c^	0.001
	IL-1β (ng/L)	21.25 ± 1.17^A^	17.53 ± 1.06^B^	14.22 ± 1.78^C^	12.33 ± 1.84^C^	< 0.0001
	IL-6 (ng/L)	23.15 ± 0.41^A^	17.66 ± 0.45^B^	16.35 ± 0.63^C^	14.22 ± 0.62^D^	< 0.0001
	IgA (μg/mL)	9.86 ± 0.53^A^	8.21 ± 0.42^B^	6.54 ± 0.91^C^^a^	5.32 ± 0.94^C^^b^	< 0.0001
	IgG (μg/mL)	94.55 ± 4.19^A^	84.35 ± 4.48^B^	79.87 ± 4.70^BC^^a^	73.27 ± 5.42^C^^b^	< 0.0001
	IgM (ng/mL)	5.76 ± 0.32^A^^a^	5.52 ± 0.36^A^^ab^	5.21 ± 0.27^AB^^b^	4.62 ± 0.38^B^^c^	0.000
60	IFN-α (pg/mL)	175.89 ± 10.12^A^	150.32 ± 9.72^B^	142.17 ± 11.35^BC^^a^	125.82 ± 12.37^C^^b^	< 0.0001
	IL-1β (ng/L)	20.24 ± 1.23^A^	16.54 ± 2.24^B^	12.33 ± 1.42^C^	11.24 ± 1.55^C^	< 0.0001
	IL-6 (ng/L)	21.55 ± 0.56^A^	16.83 ± 0.43^B^	15.69 ± 0.47^C^	13.22 ± 0.41^D^	< 0.0001
	IgA (μg/mL)	9.22 ± 0.43^A^	6.47 ± 0.25^B^	5.32 ± 0.38^C^^a^	4.81 ± 0.36^C^^b^	< 0.0001
	IgG (μg/mL)	93.18 ± 4.56^A^	82.15 ± 4.65^B^	76.55 ± 5.16^BC^	72.32 ± 5.26^C^	< 0.0001
	IgM (ng/mL)	5.55 ± 0.42^A^	5.21 ± 0.32^AB^	4.82 ± 0.25^BC^^a^	4.27 ± 0.32^C^^b^	0.000

Abbreviations: IFN-α, interferon-α; IL-1β, interleukin-1β; IL-6, interleukin-6; IgA, immunoglobulin A; IgG, immunoglobulin G; IgM, immunoglobulin M; MC20, basal diet containing 20% moldy corn; MC40, basal diet containing 40% moldy corn; MC60, basal diet containing 60% moldy corn.

Results are represented as mean values ± SD (n = 15 per group).

a,b,c Mean value within a role with no common superscript differ significantly (*P* < 0.05).

A,B,C,D Mean value within a role with no common superscript differ very significantly (*P* < 0.01).

### Effects of Moldy Corn on the Metabolism of Laying Hens

At d 20, the MC40 and MC60 contributed to the increase of AST activity (*P* < 0.01). The ALT activity and TG content were only increased in the MC60 group (*P* < 0.05). The contents of TP and HDL-C were decreased in the MC40 and MC60 groups (*P* < 0.05). The LDL-C content was reduced in the MC60 group compared to the Control and MC20 groups (*P* < 0.01). Moreover, all the moldy corn treatments decreased the T-CHO content (*P* < 0.01) and increased the UA content (*P* < 0.05).

At d 40, the AST activity and TP content were increased, while HDL-C and TG contents were decreased in the MC40 and MC60 groups (*P* < 0.05). The ALT activity and T-CHO content were increased by all the moldy corn treatments (*P* < 0.05). Besides, all the moldy corn treatments elevated the UA content (*P* < 0.01), which reached the highest in the MC60 group.

At d 60, the AST activity was increased by all the moldy corn treatments (*P* < 0.01). The ALT activity, TG content and UA concentration were increased, while LDL-C content was decreased in the MC40 and MC60 treatments (*P* < 0.05). On the contrary, the TP, HDL-C, and T-CHO contents were decreased by all the moldy corn treatments (*P* < 0.05; Table 6).

**Table 6:** Effects of moldy corn on the metabolism of laying hens.

days	Items	Treatments				*P* value
		Control	MC20	MC40	MC60	
20	AST (U/L)	28.32 ± 1.25^C^	29.89 ± 1.38^BC^	31.10 ± 1.35^AB^	32.45 ± 1.05^A^	0.001
	ALT (U/L)	3.58 ± 0.25^b^	3.54 ± 0.35^b^	3.97 ± 0.49^ab^	4.15 ± 0.21^a^	0.031
	TP (mg/mL)	52.89 ± 0.80^a^	51.96 ± 1.41^ab^	51.01 ± 1.35^bc^	50.11 ± 1.25^c^	0.015
	HDL-C (mmol/L)	1.60 ± 0.14^a^	1.55 ± 0.12^ab^	1.42 ± 0.14^bc^	1.35 ± 0.11^c^	0.024
	LDL-C (mmol/L)	0.64 ± 0.06^A^	0.64 ± 0.03^A^	0.56 ± 0.09^AB^	0.50 ± 0.05^B^	0.006
	T-CHO (mmol/L)	2.98 ± 0.07^A^	2.66 ± 0.08^B^^a^	2.67 ± 0.12^B^^a^	2.52 ± 0.09^B^^b^	< 0.0001
	TG (mmol/L)	18.25 ± 1.13^b^	18.31 ± 2.11^b^	18.88 ± 1.24^b^	20.91 ± 0.97^a^	0.031
	UA (µmol/L)	280.26 ± 8.54^B^^c^	296.89 ± 13.55^AB^^b^	310.22 ± 6.54^A^^ab^	316.04 ± 9.82^A^^a^	0.000
40	AST (U/L)	31.24 ± 1.43^b^	33.64 ± 1.75^ab^	34.10 ± 1.69^a^	35.20 ± 2.72^a^	0.036
	ALT (U/L)	4.11 ± 0.12^B^	4.41 ± 0.11^A^^b^	4.54 ± 0.08^A^^ab^	4.67 ± 0.21^A^^a^	< 0.0001
	TP (mg/mL)	54.01 ± 1.34^A^	52.33 ± 1.26^A^	48.01 ± 2.24^B^	47.06 ± 1.85^B^	< 0.0001
	HDL-C (mmol/L)	2.11 ± 0.28^a^	2.02 ± 0.28^ab^	1.78 ± 0.13^bc^	1.69 ± 0.10^c^	0.022
	LDL-C (mmol/L)	0.68 ± 0.05^a^	0.65 ± 0.04^a^	0.64 ± 0.10^a^	0.54 ± 0.03^b^	0.014
	T-CHO (mmol/L)	4.21 ± 0.18^A^^a^	3.92 ± 0.08^A^^b^	3.94 ± 0.22^A^^b^	2.84 ± 0.18^B^	< 0.0001
	TG (mmol/L)	19.66 ± 1.18^b^	20.02 ± 1.24^b^	22.17 ± 2.12^a^	22.69 ± 1.47^a^	0.014
	UA (µmol/L)	288.12 ± 3.28^C^^b^	300.97 ± 5.32^BC^^a^	302.15 ± 9.24^B^	316.89 ± 8.53^A^	< 0.0001
60	AST (U/L)	35.15 ± 1.66^B^	38.32 ± 1.76^A^	38.45 ± 1.05^A^	38.69 ± 1.55^A^	0.006
	ALT (U/L)	5.08 ± 0.34^B^^c^	5.32 ± 0.11^B^^bc^	5.48 ± 0.11^AB^^b^	5.86 ± 0.38^A^^a^	0.002
	TP (mg/mL)	53.64 ± 0.83^A^^a^	51.77 ± 1.54^A^^b^	49.13 ± 1.12^B^	48.06 ± 1.23^B^	< 0.0001
	HDL-C (mmol/L)	2.34 ± 0.08^A^	2.11 ± 0.07^B^^a^	2.04 ± 0.06^B^^ab^	1.97 ± 0.08^B^^b^	< 0.0001
	LDL-C (mmol/L)	1.05 ± 0.06^a^	0.96 ± 0.08^ab^	0.85 ± 0.22^bc^	0.74 ± 0.11^c^	0.011
	T-CHO (mmol/L)	3.82 ± 0.12^A^	3.41 ± 0.15^B^^a^	3.41 ± 0.18^B^^a^	3.11 ± 0.21^B^^b^	< 0.0001
	TG (mmol/L)	21.52 ± 0.55^b^	22.65 ± 0.93^ab^	23.48 ± 1.42^a^	23.56 ± 0.83^a^	0.016
	UA (µmol/L)	305.79 ± 4.05^C^	315.56 ± 8.69^BC^	320.24 ± 8.40^B^	335.61 ± 7.62^A^	< 0.0001

Abbreviations: AST, aspartate transaminase; ALT, alanine transaminase; TP, total protein; HDL-C, high density lipoprotein cholesterol; LDL-C, low density lipoprotein cholesterol; MC20, basal diet containing 20% moldy corn; MC40, basal diet containing 40% moldy corn; MC60, basal diet containing 60% moldy corn; T-CHO, total cholesterol; TG, total triglycerides; UA, uric acid.

Results are represented as mean values ± SD (n = 15 per group).

a,b,c,d Mean value within a role with no common superscript differ significantly (*P* < 0.05).

A,B,C Mean value within a role with no common superscript differ very significantly (*P* < 0.01).

### Effects of Moldy Corn on the Residues of Mycotoxins in Eggs

At d 20, the AFB_1_ residue in eggs were increased in the MC40 and MC60 groups (*P* < 0.01) in a dose-dependent manner. Moreover, there were no ZEN and DON residues in eggs of all the moldy corn treatment groups.

At d 40, the AFB_1_ residue in eggs were found in the MC40 and MC60 groups. The ZEN residue was only found in the eggs of the MC60 group. There were no detectable DON residues in eggs of all the moldy corn treatment groups.

**Figure 2:**
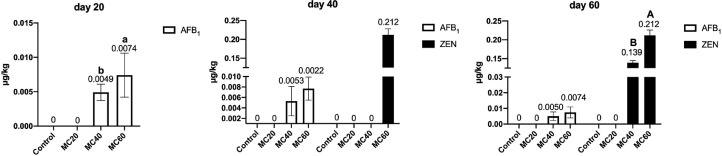
Effects of moldy corn on the mycotoxin contents in eggs. Values are means with their SD represented by vertical bars (n = 5 per group). End-point means without a common letter are significantly different (a or b, *P* < 0.05; A or B, *P* < 0.01).

At d 60, no AFB_1_ and ZEN were found in the Control and MC20 groups, but the AFB_1_ and ZEN residues in eggs were increased in the MC40 and MC60 groups, and the ZEN content in the MC60 group was higher than that of the MC40 group (*P* < 0.01). There were no DON residues in eggs of all the moldy corn treatment groups (Figure 2).

### Effects of Moldy Corn on the Residues of Mycotoxins in Muscle and Edible Viscera

**Figure 3:**
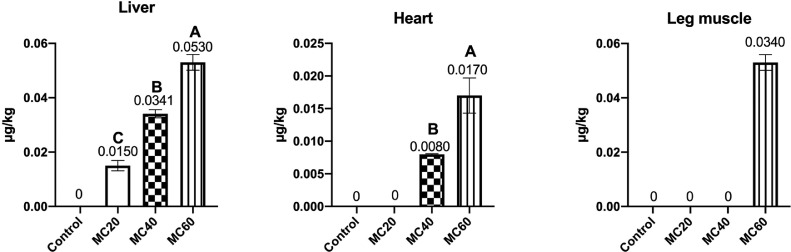
Effects of moldy corn on the AFB_1_ contents in muscle and edible viscera of laying hens. Values are means with their SD represented by vertical bars (n = 15 per group). End-point means without a common letter are significantly different (A, B or C, *P* < 0.01).

**Figure 4:**
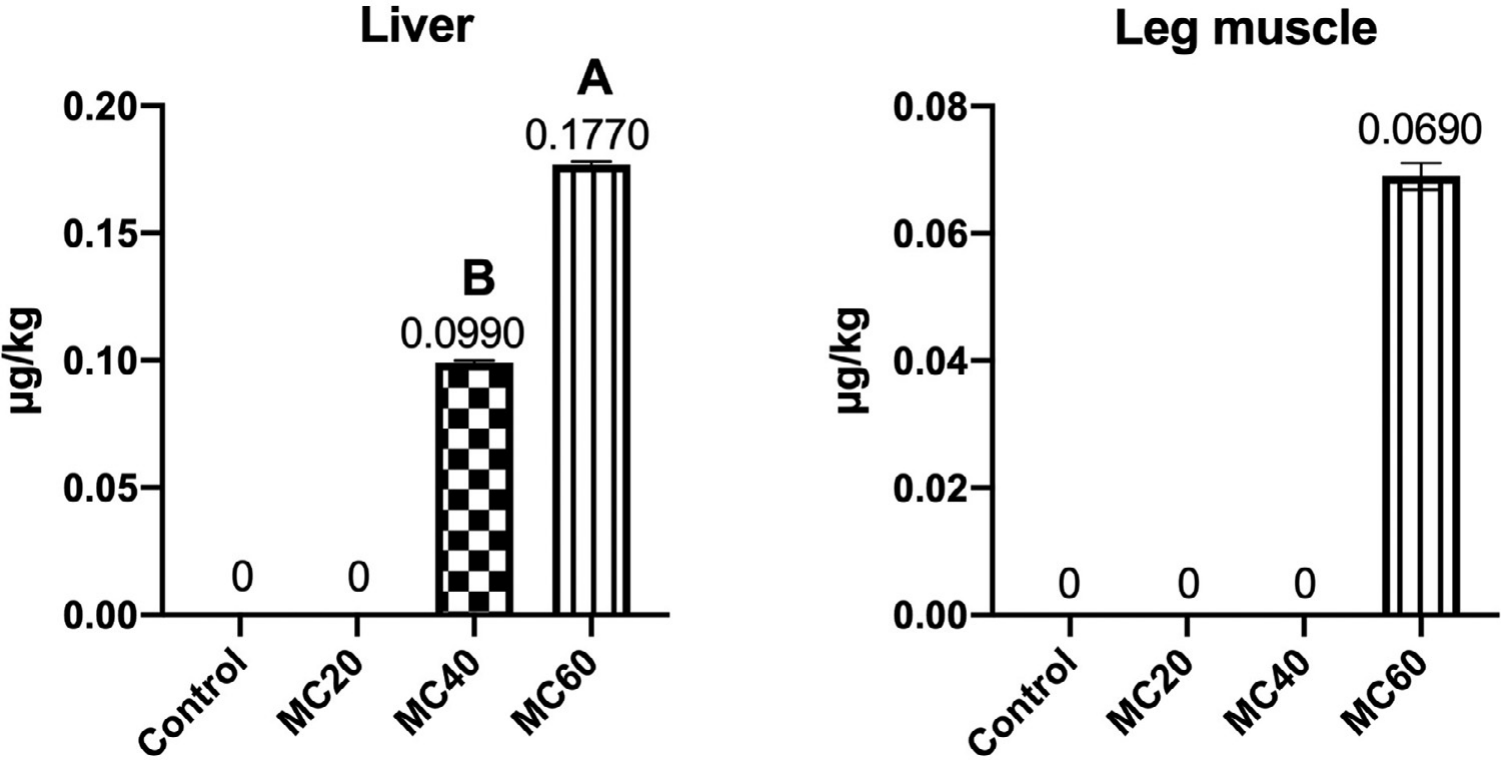
Effect of moldy corn on ZEN contents in muscle and edible viscera of laying hens. Values are means with their SD represented by vertical bars (n = 15 per group). End-point means without a common letter are significantly different (A, B or C, *P* < 0.01).

According to Figure 3, AFB_1_ residues in liver were increased in all the moldy corn treatments in a dose-dependent manner. In heart, the AFB_1_ residue was increased in the MC40 and MC60 groups (*P* < 0.01) in a dose-dependent manner. Besides, the AFB_1_ was only found in the leg muscle of MC60 group. Moreover, in the moldy corn treatments, the ZEN residues were only detected in liver and leg muscle. The MC40 and MC60 treatments increased ZEN residues in the liver in a dose-dependent manner (*P* < 0.01). The MC60 treatment also led to increased ZEN residues in leg muscle (Figure 4). Furthermore, the DON was not found in all the tested muscle and edible viscera with moldy corn treatments.

## DISCUSSION

Dietary mycotoxins in laying hens can precipitate egg production defects, leading to heavy economic losses. In China, co-contamination with AFB_1_, ZEN, and DON was commonly found in feed and feed ingredients (Ma et al., 2018). The knowledge of single toxin mechanisms of action may help predicting the potential interactive effects of mixtures, but the relative concentration of each toxin in the mixture may influence the combined final effect (Tavares et al., 2013). Considering that naturally contaminated feed could contain multiple toxins, it is important to research the toxicity and clinical signs when more than one mycotoxin is present (Christofidou et al., 2015; Jia et al., 2016).

In the present study, the AFB_1_ content in the MC20 group, the AFB_1_ and ZEN contents in the MC40 groups, and the AFB_1_, ZEN, DON contents in the MC60 group were higher than the limit standard. Although relatively high percentages of moldy corn were used in the present study, the levels of amino acids and vitamins in the 5% premix fully meet the requirements of laying hens. In addition, previous studies implied that mycotoxins, such as DON, had no significant influence on the nutrient composition of feed materials (Pietsch et al., 2014; Holanda and Kim, 2020). Bartov et al. (1982) also reported that the fat concentration of wetted whole corn did not change during storage, and no significant changes in fatty acid composition or in vitamin E, carotenoids, or protein concentration were observed. Thus, the toxic effect of moldy corn on laying hens can be considered as the consequences of toxic effects of mycotoxins.

The effects of moldy corn on the performance and health of laying hens were first investigated. Results showed that the moldy corn treatments decreased the ADFI, average egg weight, laying rate, and increased broken egg rate. Moreover, low percentage of moldy corn (MC20) could cause significant changes of ADFI and laying rate. Higher level of moldy corn (MC40 or MC60) could alter the average egg weight and broken egg rate significantly at d 20 and d 40. Moldy corn treatments also led to higher feed/egg ratio at d 40 and d 60. No death/culling occurred at d 20 and d 40, but occurred in the MC40 and MC60 groups at d 60, implying there was an additive effect of higher levels of AFB_1_, ZEN and DON on the reduction of performance. Using naturally contaminated diets containing similar AF and ZEN levels (AF: 123.0 μg/kg, ZEN: 260.2 μg/kg), Jia et al. (2016) found that naturally contaminated diets also led to lower egg production, feed intake and shell thickness of laying hens. Zhao et al. (2021) reported that dietary supplementation of 0.15 mg/kg AFB_1_, 1.5 mg/kg DON, and 0.12 mg/kg OTA decreased total feed intake, total egg weight, and egg laying rate, but increased feed/egg ratio and mortality, and the increased mortality may be due to the toxicity of OTA and higher AFB_1_ concentration than that of our study.

Mycotoxins can evoke oxidative stress by generating reactive oxygen species (**ROS**). The main consequences of ROS induced by mycotoxins, such as AFB_1_, DON, and ZEN, are damage to DNA and mitochondrial lesions (Liu and Wang, 2016; Da Silva et al., 2018). Mycotoxins can change the intracellular antioxidant mechanisms such as Nrf2, SOD, GSH-PX, and CAT expressions, inhibiting antioxidase activity and lead to increase in lipid peroxidation, which is evaluated through the amount of MDA (Da Silva et al., 2018). It has been reported that chickens fed with 100 mg/kg of AFB_1_ had significantly decreased antioxidant enzyme activities of total SOD, CAT, GSH-Px, and T-AOC but increased MDA content (Guo et al., 2021). Naturally contaminated diet also caused lower SOD activity and higher MDA level in the chicken serum (Christofidou et al., 2015). In vitro studies also displayed that various mycotoxins increased lipid peroxidation, and decreased levels of antioxidase (Lautert et al., 2014; Azizi et al., 2021). Similarly, here we also found that moldy corn treatments induced obvious oxidative stress by decreasing T-AOC, SOD, GSH-Px, and increasing MDA level at d 20, 40, and 60. Moreover, the activities of GSH-Px and SOD were sensitive to low level of moldy corn as they were significantly altered by MC20 treatment. Moreover, the MDA level was increased in the moldy corn treatments in a dose-dependent manner. Our results were consistent with previous study indicating that ZEN or DON displayed synergistic effects with AFB_1_ on oxidative damage (Lei et al., 2013).

Studies have demonstrated that immunosuppressive level elevated under mycotoxin contaminations (Berek et al., 2001; Awad et al., 2013). Mycotoxin-induced immunosuppression may be established as depressed T or B lymphocyte activity, suppressed immunoglobulin and antibody production, reduced complement or interferon activity, and impaired macrophage/neutrophil-effector activities. Besides, the inhibition of DNA, RNA, and protein synthesis via a variety of different mechanisms appears to be directly or indirectly responsible for the immunosuppressive action of many mycotoxins (Corrier, 1991). The immunosuppressive potency of various mycotoxins differs substantially. In the study by Berek et al. (2001), among the mycotoxins tested, T-2 toxin, fusarenon X, nivalenol, and DON showed the highest immunosuppressing effect in vitro. Studies in chickens also suggested that DON in feed suppressed the antibody to infectious bronchitis vaccine and Newcastle disease virus, and decreased plasma TNF-α (Awad et al., 2013); ZEN also decreased IL-6 levels in splenic lymphocytes (Wang et al., 2012). In the current study, laying hens fed with moldy corn showed decreased IFN-α, IL-1β, IL-6, IgA, IgG, and IgM concentrations. Moreover, IFN-α, IL-1β, IgG were significantly altered by MC20 treatment at d 20, but IgM was only significantly altered by MC60 at d 20, and by MC40 and MC60 for longer days, implying IgM was less sensitive to moldy corn than other parameters. Additionally, when more mycotoxins in the moldy corn were above the limit standard, the immunosuppressive effect was additive. Previous study also suggested that combined exposures to alternariol, ZEN, and DON affected the immune function of THP-1 cells additively (Solhaug et al., 2016), unfortunately, the combined toxicological effects of AFB_1_, ZEN and DON on immunity were not investigated in chickens in other studies.

Mycotoxins are hepatotoxicity, the levels of serum ALT and AST were increased in chickens fed with mycotoxin contaminated diet ( Faixová et al., 2010; Gholami-Ahangaran et al., 2016). Similarly, the moldy corn treatments in the present study also elevated the activities of ALT and AST. The liver plays a key role in lipid metabolism. Therefore, we further investigated the lipid metabolism. In the present study, the T-CHO content was decreased, while TG content was elevated by moldy corn treatments at day 20, 40 and 60. These findings were in line with the results by Zhang, in which 25 μg/kg AFB_1_ in feed increased TG (+12.5%) and decreased T-CHO (−14.17%) in the chicken blood compared to the control group (Zhang, 2018). Besides, the present data demonstrated that the TP level was decreased by moldy corn treatments. Decreased TP level was also observed in chickens fed mycotoxin-contaminated diet (Klapáčová et al., 2011; Gholami-Ahangaran et al., 2016). Moreover, the HDL-C and LDL-C contents were lowered by moldy corn treatment at days 20, 40 and 60. Similar to our results, Raei et al. (2022) reported that AFB_1_ contamination in diet also significantly reduced the HDL-C in chickens. UA is a modulator of lipid metabolism, which can affect fat accumulation through the stimulation of oxidative stress (Lima et al., 2015). Contaminated grains in broilers caused significant linear increases in serum UA concentration (Swamy et al., 2002). In the current study, moldy corn treatments elevated UA concentration at days 20, 40 and 60. It is reported that mixtures of ZEN or FB_1_ and DON display synergistic effects in lipid metabolism (Kouadio et al., 2007). Here, the lipid metabolism parameters, such as TG and TP were lower in MC40 group than that in MC20 group, suggesting that higher level of ZEN and AFB_1_ may has synergistic effects.

Mycotoxins can diffuse into foods and accumulate in the fat parts (Herzallah, 2009). In eggs, milk and meat products, different kinds of mycotoxins have been found (Herzallah, 2009; Qu et al., 2019). Low transmission rates of mycotoxin (below 1%) from feed to eggs are often reported (Sypecka et al., 2004; Meerpoel et al., 2020). Oliveira et al. (2000) investigated effects of 100, 300, or 500 μg/kg AFB_1_ in feed on the AFB_1_ residue in eggs, and found that AFB_1_ residues were detected only in eggs of hens given 500 μg/kg AFB_1_, at levels that ranged from 0.05 to 0.16 μg/kg. Previous study also reported relative lower level of AFB_1_ in the examined egg samples (Fernández et al., 1994). Similar results were obtained in the present study, demonstrating that 25.36 to 76.5 μg/kg AFB_1_ in diets led to 0.0049 to 0.0077 μg/kg AFB_1_ residue in eggs. To our knowledge, there is no fixed maximum limits for AFB_1_ residues in chicken eggs. However, Pourelmi et al. (2013) stipulated a limit of 12 ng/mL AFB_1_ for table eggs. The European Union (**EU**) also has set maximum levels for AFB_1_ and total AFs in different foods for direct human consumption, ranging from 2 to 15 μg/kg for AFB_1_ and total AFs, respectively (Ebrahem et al., 2014). The AFB_1_ contaminations in our study were lower than the aforementioned egg residuals, and none of the samples exceeded the limits. Moreover, using diets containing 5, 7.5, and 10 mg/kg DON together with 137.5, 206, and 275 μg/kg ZEN, Sypecka et al. (2004) found no ZEN residue in eggs. Inconsistently, in the present study, the ZEN was detected in eggs in the MC40 and MC60 groups. This result may be caused by the higher ZEN contents in the MC40 (505.73 μg/kg) and MC60 (750.3 μg/kg) diets. The European committee has regulated the maximum levels of ZEN ranging between 20 and 100 ppb in various food commodities (Richard, 2007), which was higher than the ZEN level in eggs of the present study. Moreover, the DON residues were not detectable in all groups at different days in this study. Similarly, previous studies also showed that DON was not detectable in eggs after chickens were fed a diet contaminated with DON (El-Banna et al., 1983; Valenta and Dänicke, 2005). The above findings indicate that the transmission rate of AFB_1_ from moldy feed to egg is low, and ZEN residue can not be detected in eggs when the ZEN content is lower than that in the MC20 group (245.05 μg/kg). Moreover, levels of DON lower than that in the MC60 diet (3.34 mg/kg) may be too low for it to be detected in eggs.

Mycotoxins in muscle and edible viscera may also damage human health. Previous study showed that the AFB_1_ residue levels in liver of chickens fed 1,600 μg/kg AFB_1_ ranged from 0.83 to 3.51 ng/g (Hussain et al., 2010). In the present study, 25.36, 51.62, and 76.5 μg/kg AFB_1_ in the MC20, MC40, and MC60 diets led to 0.015, 0.0341, and 0.053 μg/kg liver AFB_1_ residue, and the transmission rate was consistent with the study by Hussain et al. (2010). The AFB_1_ residue levels in muscles of young chicks fed 6,400 μg/kg AFB_1_ was 3.27 ng/g, and the transmission rate of AFB_1_ from feed to liver was higher than our result. This may be due to the combination of multiple mycotoxins, because the combined effects of mycotoxins in feed resulted in increased residues and in broiler chickens’ organs (Chang et al., 2020). Moreover, the Food and Drug Administration (**FDA**) tolerance level of AF in human food is 20 μg/kg (El-Nabarawy et al., 2020), which was higher than the detected AFB_1_ levels in muscle and edible viscera of the present study. Due to the global occurrence of ZEN, the European Food Safety Authority (**ESFA**) has set guidelines for the maximum levels of ZEN allowable in various foodstuffs (Thapa et al., 2021), which were also higher than the detected ZEN levels in liver and leg muscle in the present study. Moreover, report showed that 2.5 mg/kg ZEN in feed led to around 1,100 ng/kg ZEN residue in liver (Jia et al., 2022), and the transmission rate of ZEN from feed to liver was a little higher than that in our study. This may be due to the high ZEN metabolism in chickens (Tardieu et al., 2021), as Dänicke et al. (2002) reported that α-ZEL was detectable in the liver, whereas ZEN was not detected in either breast meat or the liver of laying hens. However, the ZEN metabolism should be further investigated.

In conclusion, the moldy corn with excessive AFB_1_, ZEN, DON above the limits can impair the ADFI and laying performance of laying hens. Moreover, the moldy corn also decreased antioxidant capacity, increased immunosuppression, impaired liver function and metabolism. Although AFB_1_ and ZEN residues were found in eggs, muscle and edible viscera, the residue levels were relatively low and may not have adverse impact on human health. In addition, the toxic effects and mycotoxin residues (AFB_1_ and ZEN) were increased with the increase of moldy corn levels in feed. However, the combination effects of multiple mycotoxins including AFB_1_, ZEN and DON, should be further explored by using binary and ternary mixtures.

## Appendix Supplementary materials

Image, application 1

## Footnotes

Supplementary material associated with this article can be found in the online version at doi:10.1016/j.psj.2023.102502.
