# Supplementary material for: Effects of moldy corn on the performance, antioxidant capacity, immune function, metabolism and residues of mycotoxins in eggs, muscle, and edible viscera of laying hens
Source: Poult Sci. 2023 Jan 14;102(4):102502. doi: 10.1016/j.psj.2023.102502 (PMC9932114; doi:10.1016/j.psj.2023.102502)
Supplement: Supplementary file 1 [file mmc1.docx]

Supplementary Material

**Table S1.** **Multiple reaction monitoring (MRM) data-acquisition parameters for mycotoxins**

| Mycotoxins | Precursor ion  (m/z) | Product ion  (m/z) | Cone voltage  (V) | Collision energy  (eV) | Peak time  (s) |
| --- | --- | --- | --- | --- | --- |
| AFB_1_ | 313＞214 | 313＞241 | 180 | 35 | 3.8 |
| DON | 341＞247 | 341＞265 | 90 | 6 | 0.5 |
| ZEN | 319＞160 | 319＞275 | 100 | 20 | 5.1 |

**Table S2.** **Performance characteristics for the analysis of the targeted mycotoxins in egg and liver**

| Sample | Mycotoxins | Concentration (μg/kg) | Recovery range (%) | RSD (%) |
| --- | --- | --- | --- | --- |
| Egg | AFB_1_ | 5 | 80.5~92.7 | 4.6 |
|  |  | 20 | 81.3~94.5 | 4.9 |
|  |  | 40 | 85.5~96 | 4.7 |
|  | ZEN | 20 | 97.3~106.8 | 4.2 |
|  |  | 40 | 96~107.6 | 5.1 |
|  |  | 80 | 94.2~104.3 | 4.8 |
|  | DON | 100 | 80.7~92.2 | 5.0 |
|  |  | 400 | 82.5~95.5 | 4.8 |
|  |  | 800 | 85~101.6 | 6.3 |
| Liver | AFB_1_ | 5 | 87.5~96.7 | 4.6 |
|  |  | 20 | 89~98.5 | 4.5 |
|  |  | 40 | 85.5~95 | 4.7 |
|  | ZEN | 20 | 94~105 | 4.8 |
|  |  | 40 | 92.1~104.6 | 5.5 |
|  |  | 80 | 91.5~102 | 4.3 |
|  | DON | 100 | 83~95.6 | 5.7 |
|  |  | 400 | 85.6~98 | 6.0 |
|  |  | 800 | 88~103.1 | 6.5 |
| Heart | AFB_1_ | 5 | 86.5~96.3 | 4.4 |
|  |  | 20 | 82~97.5 | 5.6 |
|  |  | 40 | 85.8~99.6 | 5.2 |
|  | ZEN | 20 | 93.6~105.3 | 4.9 |
|  |  | 40 | 91~102 | 4.6 |
|  |  | 80 | 90.5~102.7 | 4.5 |
|  | DON | 100 | 80~93.6 | 5.2 |
|  |  | 400 | 82.6~95 | 6.1 |
|  |  | 800 | 84~96.1 | 6.0 |
| Breast muscle | AFB_1_ | 5 | 82~96.6 | 5.6 |
|  |  | 20 | 85.5~98 | 5.1 |
|  |  | 40 | 93.7~102 | 6.3 |
|  | ZEN | 20 | 99~110.4 | 4.4 |
|  |  | 40 | 97.3~108.2 | 4.3 |
|  |  | 80 | 92~105.3 | 4.9 |
|  | DON | 100 | 88.2~101.5 | 5.1 |
|  |  | 400 | 90.6~103.2 | 5.5 |
|  |  | 800 | 87.1~98.9 | 4.6 |
| Leg muscle | AFB_1_ | 5 | 85.7~96.3 | 4.7 |
|  |  | 20 | 85.1~95.5 | 4.5 |
|  |  | 40 | 87.7~99.9 | 5.0 |
|  | ZEN | 20 | 97.6~111 | 5.9 |
|  |  | 40 | 98.3~112.7 | 6.0 |
|  |  | 80 | 97.6~110 | 6.2 |
|  | DON | 100 | 85.17~98.6 | 5.4 |
|  |  | 400 | 89~101.6 | 5.5 |
|  |  | 800 | 91.2~105.3 | 6.1 |

**Table S3.** **LOD and LOQ for the analysis of the targeted mycotoxins in egg and viscera**

| Mycotoxins | LOD (μg/kg) | LOQ (μg/kg) |
| --- | --- | --- |
| AFB_1_ | 0.0015 | 0.0045 |
| ZEN | 0.06 | 0.20 |
| DON | 2.7 | 9.13 |
